# Multiple Responsive Hydrogel Films Based on Dynamic Phenylboronate Bond Linkages with Simple but Practical Linear Response Mode and Excellent Glucose/Fructose Response Speed

**DOI:** 10.3390/polym15091998

**Published:** 2023-04-23

**Authors:** Rong Xu, Jiafeng Tian, Yusheng Song, Shihui Dong, Yongjun Zhang

**Affiliations:** 1 China Academy of Aviation Manufacturing Technology, Beijing 100024, China; 2 School of Chemistry, Tiangong University, Tianjin 300387, China

**Keywords:** multiple responsive, dynamic phenylboronate bond, layer-by-layer

## Abstract

Multiple responsive hydrogels are usually constructed by the addition of many different functional groups. Generally, these groups have different responsive behaviors which lead to interleaved and complex modes of the multi-response system. It is difficult to get a practical application. In this study, we show that multi-response hydrogels can also be constructed using dynamic bonds as crosslinks. The multiple responsive hydrogel films with thicknesses on the sub-micrometer or micrometer scale can be fabricated from P(DMAA-3-AAPBA), a copolymer of N,N-dimethylacrylamide, 3-(acrylamido)phenylboronic acid, and poly(vinylalcohol) (PVA) though a simple layer-by-layer (LbL) technique. The driving force for the film build up is the in situ-formed phenylboronate ester bonds between the two polymers. The films exhibit Fabry–Perot fringes on their reflection spectra which can be used to calculate the equilibrium swelling degree (SD_e_) of the film so as to characterize its responsive behaviors. The results show that the films are responsive to temperature, glucose, and fructose with simple and practical linear response modes. More importantly, the speed of which the films respond to glucose or fructose is quite fast, with characteristic response times of 45 s and 7 s, respectively. These quick response films may have potential for real-time, continuous glucose or fructose monitoring. With the ability to bind with these biologically important molecules, one can expect that hydrogels may find more applications in biomedical areas in the future.

## 1. Introduction

Stimuli-responsive hydrogels are capable of undergoing sharp and reversible volume change in response to external stimuli, such as physical (temperature, electric or magnetic fields, and mechanical stress) [1,2,3,4] or chemical (pH, metal ions, and other chemical molecules) stimuli [5,6,7]. They have been used as intelligent materials in many fields, especially in sensing [8], drug delivery [9], actuators [10], and chemical valves [11,12].

Usually, stimuli-responsive hydrogels are designed based on the introduction of a sensing element into the polymeric network [11,13,14]. For example, thermosensitive polymer poly(N-isopropylacryl-amide) (PNIPAM) was widely used to construct thermosensitive hydrogels [15,16,17]. Other sensing elements, such as carboxylic acid or amino groups, phenylboronic acid groups, and azobenzene groups, were used for pH-responsive [18], glucose-sensing [11,19,20] and light-sensing [21] hydrogels, respectively. When two or more functional groups are incorporated into an intelligent hydrogel system, hydrogels with dual responsivity or multiple responsivity are constructed [22,23]. For example, Wang et al. [24] prepared thermal and pH-responsive hydrogels with the addition of alkyltrimethylammonium bromide and sodium azobzenzene 4,40-dicarboxylic acid (AzoNa_2_). Singh et al. [25] prepared multi-stimuli-responsive (pH-, CO_2_-, and thermo-responsive) foams using an anionic surfactant with three components: a hydrophobe (tristyrylphenol), a temperature-sensitive block (polypropylene oxide), and pH-sensitive moiety (carboxyl group).

Although the simple stacking of functional monomers can achieve the goal of multiple sensor responses, it will also bring some problems, such as a complicated preparation process and complex interleaved sensing modes. For example, Ren et al. [26] designed a high-performance underwater strain hydrogel sensor. The sensor preparation process requires the incorporation of several different functional groups in the system, such as hydrogen bonding provided by flexible PHEMA chains, the formation of PVA microcrystalline structural domains during the freezing–thawing process as cross-linking points for the amorphous hydrogel network, and the inhibition of hydrogel swelling via the addition of HCl. The preparation process is very complex, making it difficult to obtain further practical applications.

The ideal hydrogel sensor should have a simple preparation process and a practical sensing mode with a fast response time [13,20]. The response time of hydrogen is directly associated with the slow (de)swelling of the hydrogel [27]. It is well known that fully (de)swelling bulky gels usually takes hours or even weeks [28]. For example, Khademhosseini et al. [29] used deep eutectic solvents (DES) instead of water or organic solvents as a medium for the photopolymerization of amphiphilic ions and polarized monomers, resulting in a new ionic bulk gel with tunable mechanical properties. The hydrogel has excellent environmental resistance but a slow response rate due to its centimeter-level hydrogel size. Stimuli-responsive hydrogels have earned a reputation as intelligent materials in the field of sensing. However, their practical applications have been hindered, largely because of their slow response [27,30]. Therefore, simple, practical, and fast-responding hydrogel sensors are highly desirable.

Dynamic bonds are chemical bonds capable of undergoing reversible breakage and reformation [31]. The tunable and reversible nature of non-covalent interactions leads to the superior properties of supramolecular hydrogels, such as stimuli-responsiveness [32,33,34] and self-healing [35,36].Stimuli-responsive hydrogel systems based on dynamic bond linkages have been studied by many researchers [14,37,38]. However, these hydrogel systems generally require complex functional group synthesis [37] and modification processes [38], and the size of the prepared hydrogel films are generally in the centimeter range with slow responses to stimuli [14,38,39], which makes it difficult to achieve rapid sensing applications. In this study, we prepared multiple responsive hydrogels constructed using dynamic bonds as crosslinks via the adoption of layer-by-layer assembly, which is a simple but versatile approach, and using hydrogel films with thicknesses on sub-micrometer or micrometer scale, which is two orders of magnitude thinner than the films used in ordinary hydrogel sensors, and can be facilely fabricated. Thanks to the ultra-thin thickness, the hydrogel films have a fast response.

In this work, a dynamic phenylboronate bond has been introduced into the multiple responsive hydrogels as crosslinks. We synthesized a series of polymers, P(DMAA-3-AAPBA), a copolymer of N,N-dimethylacrylamide, and 3-(acrylamido)phenylboronic acid. These copolymers have a similar structure but different phenylboronic acid content.

Then, copolymers were individually combined with PVA using a simple layer-by-layer (LbL) technique to prepare hydrogel films. The driving force for the film buildup was the in situ-formed phenylboronate ester bonds between the two polymers. The films exhibited Fabry–Perot fringes on their reflection spectra. Using this special optical phenomenon, the assembly process and swelling behaviors of the film were studied. The results showed that the hydrogel films exhibited good stimuli-responsive behavior to temperature, glucose, and fructose, and all of the stimuli-responsive behaviors of the hydrogel films were linearly correlated. For an ideal sensor, a linear response is highly desirable because it leads to a simple calibration and constant sensitivity and precision over the entire linear range. More importantly, the response of the hydrogel films to glucose or fructose was quite fast, making it possible for the LbL films to be used for continuous biosensing.

## 2. Materials and Methods

### 2.1. Materials

N,N-dimethylacrylamide (DMAA), acryloyl chloride, acrylic acid (AAc), 2,2′-azo-bis-isobutyronitrile (AIBN), poly(vinylalcohol) (PVA) (DP = 1750 ± 50), and other reagents were purchased from the Tianjin Chemical Reagents Company(Tianjin, China). Other purchases included 3-aminophenylboronic acid hemisulfate (APBA), (3-aminopropyl) triethoxysilane (APTES, 99%), and 1-(3-dimethyl aminopropyl)-3-ethylcarbodiimide hydrochloride (EDC) from Aladdin (Shanghai, China). According to reference [40], 3-(acrylamido)phenylboronic acid (3-AAPBA) was synthesized from acryloyl chloride and APBA. Glucose and fructose were purchased from Heowns (Tianjin, China). Other chemicals were used as received.

### 2.2. Copolymer Synthesis

The copolymer of AAPBA and DMAA (P(DMAA-3-AAPBA)) was synthesized by free radical copolymerization [20]. Briefly, the comonomers and initiator (2.0 mg of AIBN) were dissolved in 40 mL of N,N-dimethylformamide (DMF). The total concentration of monomers was 0.28 M. Molar ratios of the comonomers are listed in Table 1. The mixture was purged with nitrogen for 30 min to remove the dissolved oxygen and then heated to 80 °C to initiate free radical polymerization. The reaction was allowed to proceed under nitrogen bubbling for 12 h. The product was precipitated in acetone, filtered, washed three times with acetone, and then dried under a vacuum.

### 2.3. Film Fabrication Using the LbL Technique

Silicon wafers were used as substrates for film fabrication. The wafers were first cleaned in a boiling piranha solution (3:7 *v/v* H_2_O_2_–H_2_SO_4_ mixture), rinsed thoroughly with deionized (DI) water, and dried. To introduce amino groups, they were immersed in a 1 wt% toluene solution of APTES overnight, washed in toluene for two minutes, and dried at 120 °C in an oven. A layer of polyacrylic acid (PAAc) was first assembled by immersing the substrates in a 0.1 wt% aqueous solution of PAAc (pH 3.0) for 10 min, followed by washing it with DI water. PBA groups were introduced by treating the substrates in an aqueous solution containing 7.5 mM APBA and 12.5 mM EDC for 4 h. To assemble the LbL films, the substrates were then immersed in 0.2 wt% solutions of PVA and P(DMAA-3-AAPBA) (in a 50 mM, pH 8.5 phosphate buffer) alternately, each for 4 min, and intermediated with water washing. Films with various thicknesses were fabricated by repeating the deposition cycles. They were then washed with water and air-dried [20,41,42].

### 2.4. Film Swelling

The swelling of the films was studied using an experimental setup shown in Figure S1. The film was immersed in a phosphate buffer, and its reflection spectra were measured with a fiber optic spectrometer. The temperature of the sample cell was controlled with a refrigerated circulator [12,20,43].

### 2.5. Characterization

Reflection spectra of the LbL hydrogel films were measured with an AvaSpec-2048 Fiber Optic Spectrometer [12,19,41,43] with resolution up to 0.05 nm. ^1^H NMR spectra of the copolymers were recorded with a Varian UNITY-plus 400 NMR spectrometer using D_2_O as a solvent with an accuracy of 400 MHz. The molecular weight was determined with a Viscotek chromatograph (GPC max, Houston, TX, USA) using a Triple Detector Array 302 (Viscotek, Houston, TX, USA) with refractive index, viscosity, and static light scattering at 30 °C. Column set: TSK GMPWXL. Eluent: 0.2 M NaNO_3_/0.1 M NaH_2_PO_4_. Flow rate: 1.0 mL/min. The SD_e_ data of all LbL films were repeated by using three groups of data to determine the average and calculate the variance.

## 3. Results and Discussion

### 3.1. Copolymer Synthesis and Characterization

**Scheme 1:**
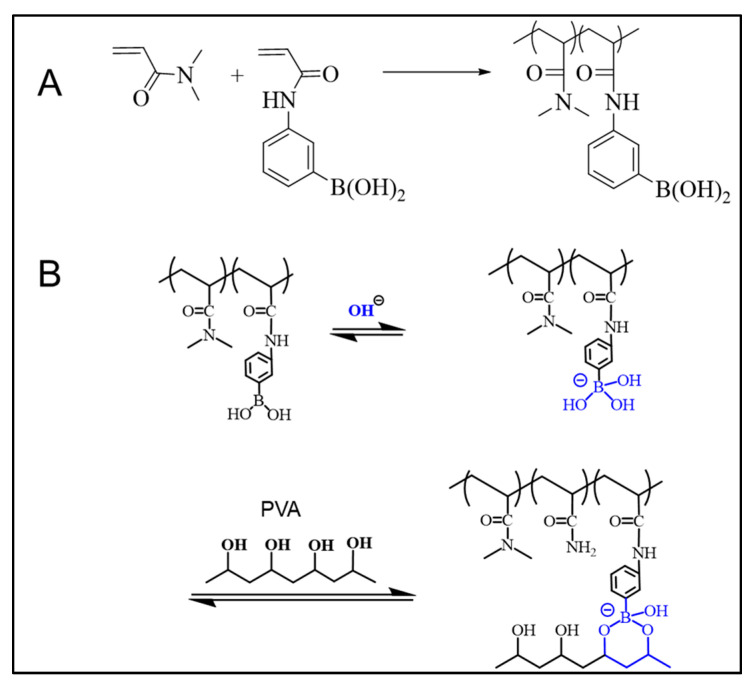
(**A**). Synthesis of P(DMAA-3-AAPBA) by free radical polymerization. (**B**) The formation of phenylboronate bond linkages between of PVA and P(DMAA-3-AAPBA) by LbL.

**Figure 1:**
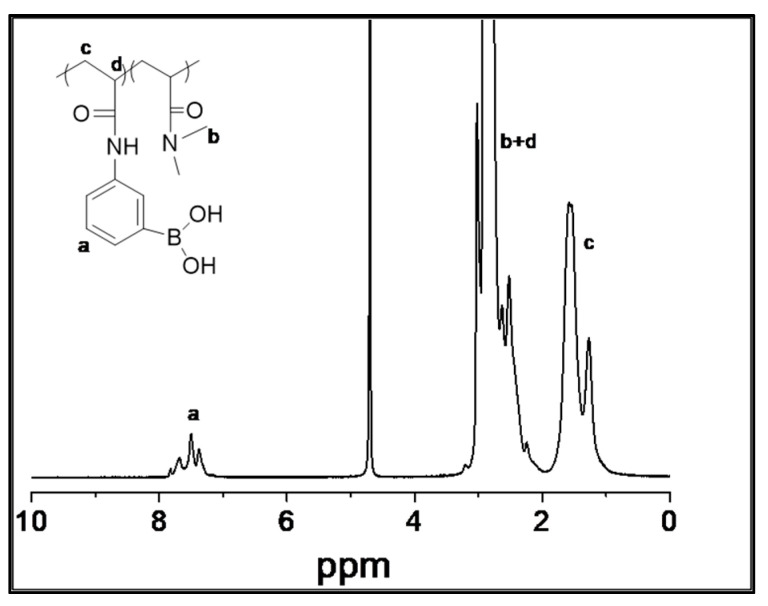
The ^1^H NMR spectra of P(DMAA-3-AAPBA) copolymers.

A series of copolymers of N,N-dimethylacrylamide (DMAA) and 3-(acrylamido) phenylboronic acid (3-AAPBA), P(DMAA-3-AAPBA) were synthesized by free radical polymerization. As shown in Figure 1, the NMR spectrum of the polymerization reaction product indicates that the product has been successfully synthesized. In these copolymers, the 3-AAPBA units act as reaction sites for the binding of PVA (Scheme 1A). These polymers are designed to contain different molar ratios of phenylboronic acid contents (3-AAPBA), with AAPBA content of 5 mol%, 10 mol%, and 15 mol%, according to the molar ratio in the feed. 

Detailed characterizations of the copolymer are listed as Entries 1–3 in Table 1. The determined copolymer compositions are close to the theoretical ones.

### 3.2. Hydrogel Film Fabrication

LbL films were fabricated by alternate immersion of a PBA-modified substrate in PVA and P(DMAA-3-AAPBA) solutions. The driving force for the film buildup is the covalent phenylboronate ester bonds formed in situ between the two polymers (Scheme 1B). The formation of phenylboronate ester bonds in LbL films from PVA and PBA-containing polymers was previously confirmed by the appearance of the marker mode of boronate ester in the IR spectra of the films [44,45].

To fabricate the hydrogel films, the substrates were first modified with the amino groups. They were then dipped into 0.2 wt% solutions of PVA and P(DMAA-3-AAPBA) (in a 50 mM, pH 8.5 phosphate buffer) alternately, and films with thicknesses of 100 bilayers were fabricated by repeating the deposition cycles. These films present an interesting optical phenomenon [46].

**Figure 2:**
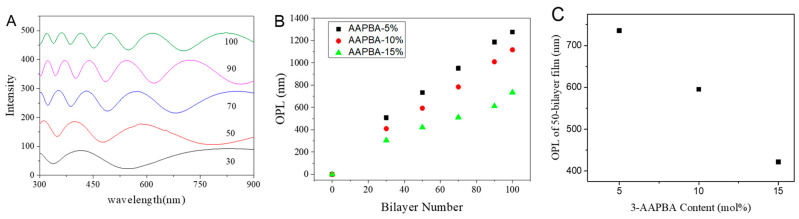
(**A**) Reflection spectra of the LbL films fabricated from PVA and P(DMAA-3-AAPBA) copolymers with various bilayer numbers as indicated. AAPBA content: 10 mol%. The films were assembled in a 50 mM, pH 8.5 phosphate buffer at 25 °C. The plots are shifted along the vertical axis for clarity. (**B**) Calculated OPL of the films as a function of the bilayer number. (**C**) OPL of 50 bilayer films as a function of 3-AAPBA content in the copolymers.

As shown in Figure 2A, the reflection spectra of the films display oscillations in the UV, visible, and near-IR range we examined. These peaks, known as Fabry–Perot fringes, stem from the interferences between beams reflected at the air–film and film–substrate interfaces [47]. Using these fringes, film growth can be facilely monitored, as the thickness θ or the optical path length (OPL) of the film can be calculated using the following relationship:

(1) $$\theta =\frac{1}{2n_{e}\left(1/\lambda_{p}-1/\lambda_{p+1}\right)}\;\mathrm{or}\;\mathrm{OPL}=n_{e}\times \theta =\frac{1}{2\left(1/\lambda_{p}-1/\lambda_{p+1}\right)}$$

where n_e_ is the refractive index and λ_p_ and λ_p+1_ are the two adjacent wavelengths for which the intensity is maximal.

As shown in Figure 2B, the calculated OPL of the films increases linearly with the bilayer number, suggesting a linear growth of the films. Linear growth has been widely reported for LbL films. Particularly, LbL films fabricated from PVA and other PBA-containing polymers were previously reported to grow linearly [20], indicating that the film growth is regular and reproducible from each layer.

Although all the copolymers assemble regularly with PVA, the film growth rate is different. Figure 2C compares the OPL of 50 bilayer films from the copolymers with different 3-AAPBA contents. When the 3-AAPBA content increases from 5 mol% to 15 mol%, the film growth rate decreases. It is likely that as the 3-AAPBA content increases, more phenylboronate ester bonds form between the copolymer and PVA, more phenylborate anion exists, and the repulsion among the anions charges is unfavourable for the film build up [48].

### 3.3. Swelling in Water

**Figure 3:**
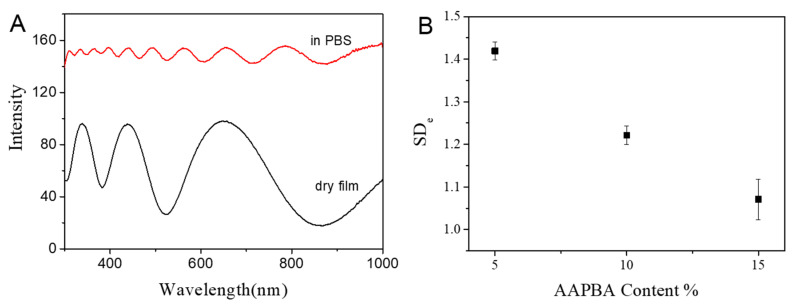
(**A**) Reflection spectra of the LbL films fabricated from PVA and P(DMAA-AAPBA) copolymer measured in air (dried film) and in water (swollen film). AAPBA content: 5 mol%. The films were assembled in a 50 mM, pH 8.5 phosphate buffer at 25 °C. The plots are shifted along the vertical axis for clarity. (**B**) SD_e_ of the films in water as a function of the 3-AAPBA content of copolymer.

Like ordinary hydrogels, the LbL hydrogel films also swell when immersed in water. In addition, they still present Fabry–Perot fringes, thus providing a facile way to study their swelling. As an example, Figure 3A shows the reflection spectra of a 100 bilayer film measured in air (dry film) and in phosphate buffer (swollen film). Compared with the spectra measured in air as dry film, more fringes appear when the film is soaked in water. OPLs of the film can be determined using Equation (1), which are 670 and 1487 nm for the dry and wet film, respectively. The results clearly indicate that the film swells when immersed in water. The equilibrium swelling degree (SD_e_) of the film was then calculated using the following equation: considering that the decrease in the refractive index of the film is negligible, where OPL_s_ and OPL_d_ are the OPL of the swollen and dry films, respectively.

(2) $${\mathrm{SD}}_{e}=\frac{{\mathrm{OPL}}_{s}-{\mathrm{OPL}}_{d}}{{\mathrm{OPL}}_{d}}$$

As shown in Figure 3B, regarding the SD_e_ of the films as a function of the 3-AAPBA content, a clear trend was observed that SD_e_ decreases with increasing 3-AAPBA content of the copolymers. This is reasonable because the swelling degree of a hydrogel decreases with an increasing crosslinking degree, and the crosslinking degree of the PVA/P(DMAA-3-AAPBA) film is supposed to increase with an increasing 3-AAPBA content of copolymer P(DMAA-3-AAPBA).

### 3.4. Thermosensitive Behavior

**Figure 4:**
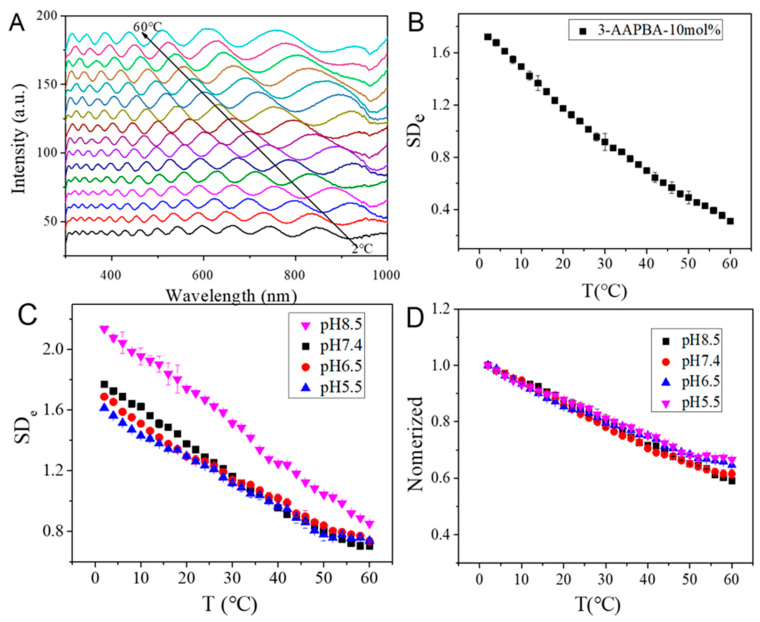
(**A**) Fabry−Perot fringes of the film shift as the glucose concentration increases. The films were soaked in a 50 mM, pH 8.5 phosphate buffer. AAPBA content is 10 mol%. (**B**) The equilibrium swelling degree (SD_e_) of the LbL films measured at various temperatures. The films were soaked in a 50 mM, pH 7.4 phosphate buffer. The films were fabricated from PVA and P(AAm-DMAA-3-AAPBA) copolymers. (**C**) SD_e_ of P(DMAA-3-AAPBA)/PVA films measured at various temperatures in different pH 50 mM PBS. (**D**) Normalized SD_e_ of P(DMAA-3-AAPBA)/PVA films measured at various temperatures in different pH 50 mM PBS. The heating rate is 2 °C/10 min, and the AAPBA content in the copolymer is 10 mol%.

Figure 4A illustrates how the film’s Fabry–Perot fringes change as the temperature rises, demonstrating significant thermosensitive behavior. The SD_e_ of the film gradually decreases and exhibites a certain linear correlation. The film’s thermosensitive behavior in various pH conditions was then investigated. The film has comparable thermosensitive behavior under various pH settings, as seen in Figure 4B. The sole distinction is that the absolute value of the SD_e_ changes depending on the pH. The thermosensitive behavior of the film differs slightly when the pH is lower than 7.4. When the pH reaches 8.5, the SD_e_ of the film suddenly increase, which is related to the PKa of the phenylborate ester bond. It has been reported in the literature that the pKa of PBA is 8.2 [11,49]. When the pH in the system is greater than 8.2, free phenylborate exist in the form of anions, and there is an anionic repulsion effect with the phenylborate anion in the LbL film, which increases the swelling degree of the film. As shown in Figure 4D, the thermosensitive behaviors of film at different pH conditions tend to converge after normalization, which also indicates that the thermosensitive behavior of the film is less affected by environmental pH. This is beneficial for future applications of the film as a temperature sensitive sensor.

### 3.5. Glucose-Sensitivity Behavior

**Figure 5:**
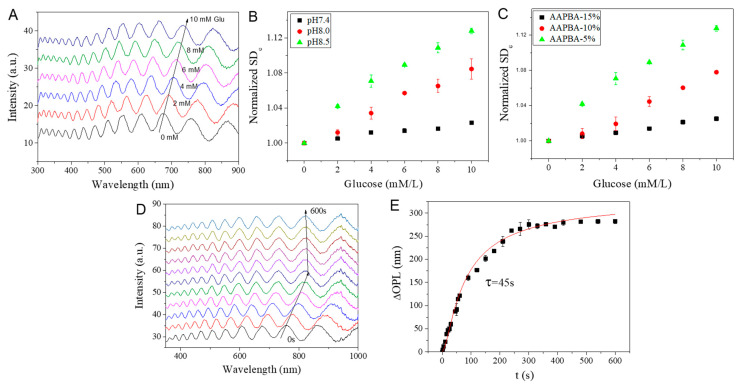
(**A**) Fabry−Perot fringes of the film shift as the glucose concentration increases. The films were soaked in 50 mM pH 8.5 phosphate buffer. AAPBA content is 5 mol%, T = 25 °C. (**B**) the variation of SD_e_ of P(DMAA-3-AAPBA)/PVA film as glucose concentration increases in different pH 50 mM PBS. AAPBA content is 5 mol%. (**C**) The variation of SD_e_ of P(DMAA-3-AAPBA)/PVA film 50 mM, pH 8.5, phosphate buffer at 25 °C with different AAPBA Content. (**D**) Fabry−Perot fringes of the film upon addition of 10 mM glucose. The data were measured in 50 mM, pH 8.5, phosphate buffer at 25 °C. (**E**) Response kinetics of a PVA/P(AAm-AAPBA) in 50 mM, pH 8.5, phosphate buffer at 25 °C upon addition of 10 mM glucose. AAPBA content is 5 mol%.

**Scheme 2:**
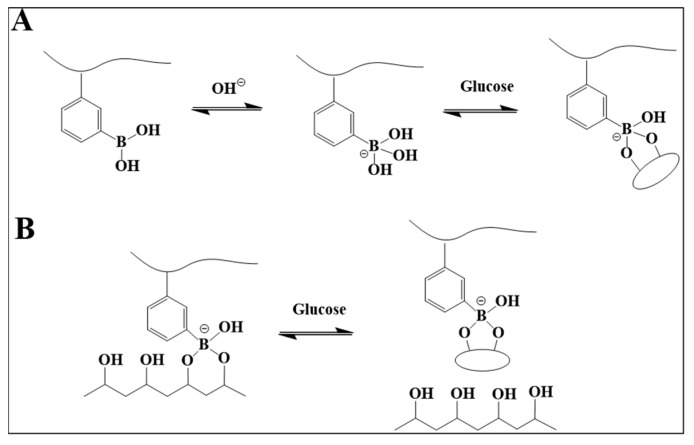
Two ways that glucose binds with PBA groups in the film: (**A**) binding with free PBA groups and (**B**) competing with PVA for the PBA binding sites.

The glucose-responsivity of the resultant PVA/P(DMAA-3-AAPBA) films was then tested. As shown in Figure 5A, Fabry−Perot fringes of the film shifted as glucose concentration increases. From the reflection spectra, the SD_e_ of the film was calculated and plotted against glucose concentration (Figure 5B). As expected, SD_e_ increased with the addition of glucose. The glucose-induced swelling of the films could be attributed to the binding of glucose with PBA groups in the films. As Scheme 2 shows, glucose may react with PBA groups in the films in two ways. It may bind with the free PBA groups and convert them from a neutral, hydrophobic form to a negatively charged, hydrophilic form. The increased charge density of the copolymer will be favorable for film swelling. More importantly, glucose may compete with PVA for the PBA binding sites. As a result, the crosslink density of the film decreases. In both ways, the binding of glucose with PBA groups in the film results in a larger swelling of the film. We also studied the effect of pH to film glucose-sensitivity. The highest sensitivity toward glucose was observed at pH 8.5, while at physiological pH a rather low glucose-sensitivity was observed. Similar pH-dependent glucose-sensitivity has been reported previously for other PBA-based glucose-sensitive materials [19,20]. The AAPBA used here is a weak acid with a pKa of 8.2 [11,49]. Both the undissociated, planar trigonal structure and the dissociated, tetrahedral structure can react reversibly with glucose; however, only the tetrahedral structure can form a stable complex [50]. As a result, the film only exhibited good glucose-sensitivity at relatively high pHs.

As shown in Figure 5C, the glucose-sensitivity of the films assembled by different contents of phenylboronic acid copolymers varies greatly. When the AAPBA content ranges from 5 mol% to 15 mol%, the variation in the SD_e_ gradually decreases, i.e., the glucose-sensitivity gradually decreases. It is reasonable to assume that the crosslink density of the film increases with the increase of AAPBA content in copolymers used for LbL films. When glucose interacts with PBA groups in the LbL films, the system with a low cross-linking degree must have a significant change in the swelling degree.

Figure 5D shows Fabry−Perot fringes of the film upon the addition of 10 mM glucose. It is obvious that the Fabry−Perot fringes shift quickly and reach balance. Figure 5E shows the swelling kinetics of the film in a 10 mM glucose solution. From the single exponential fit of the data, the characteristic swelling time, τ, i.e., the time to obtain 1/e of the total change in optical length, was determined to be only 45 s at 25 °C.

### 3.6. Fructose-Sensitivity Behavior

**Figure 6:**
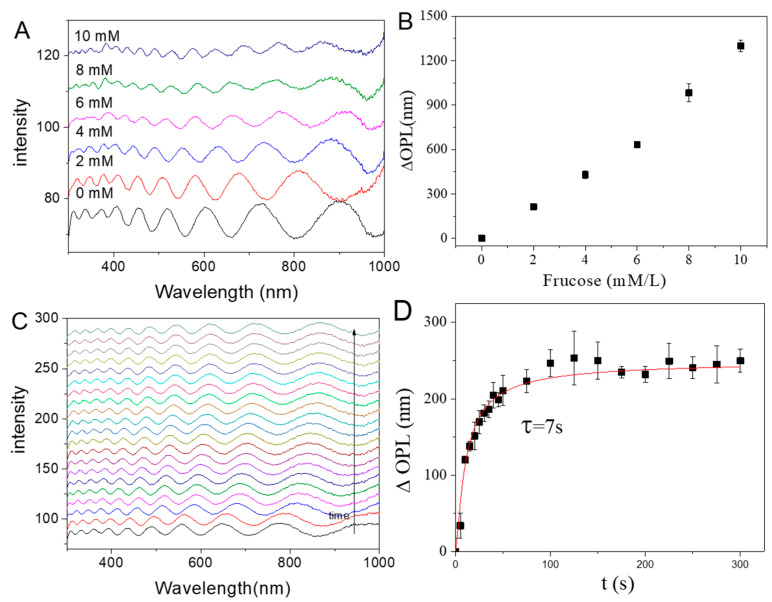
(**A**) Fabry−Perot fringes of the film shift as the fructose concentration increases. The films were soaked in a 50 mM, pH 8.5 phosphate buffer at 25 °C. AAPBA content is 5 mol%. (**B**) The ΔOPL of P(DMAA-3-AAPBA)/PVA film as the fructose concentration increases in different pH 50 mM PBS. AAPBA content is 5 mol%. (**C**) Fabry−Perot fringes of the film shift upon the addition of 2 mM fructose at 25 °C. (**D**) Response kinetics of a PVA/P(AAm-AAPBA) in a 50 mM, pH 8.5 phosphate buffer at 25 °C upon the addition of 2 mM fructose at 25 °C. AAPBA content is 5 mol%.

It is well-known that glucose, as a 1,2-diol, can bind with the PBA group to form boronated esters [51]. Fructose, as an isomer of glucose, should also be able to react with the LbL films. As seen in Figure 6A, the addition of fructose causes the Fabry–Perot fringes of the film to shift, and the degree of the shift increases as the concentration of fructose increases. The ΔOPL (nm) increases with an increasing fructose concentration (Figure 6B). That means that the film is also responsive to fructose. We also studied response kinetics of a PVA/P(AAm-AAPBA) film. As shown in Figure 6C, the Fabry−Perot fringes of the film shift rapidly and gradually reach equilibrium as time increases upon the addition of a 2 mM fructose solution. Figure 6D shows the swelling kinetics of a 100 bilayer film in 2 mM fructose. We can see that the ΔOPL (nm) of the film increases by 274 nm after the addition of 2 mM fructose. That means that the fructose induced swelling of the film. Fructose, like glucose, reacts with the phenylboronic ester linkages, substituting the matching crosslinking point, lowering the degree of crosslinking, or raising the charge density, which causes the film to swell. From the single exponential fit of the data, the characteristic swelling time, τ, i.e., the time to obtain 1/e of the total change in optical length, was determined to be only 7 s at 25 °C. From the characteristic response time, it can be seen that fructose may be more sensitive to phenylboronic acid than glucose.

## 4. Conclusions

In conclusion, we fabricated multiple-responsive hydrogel films which have a fast response speed to glucose/fructose based on the dynamic phenylboronate ester bonds. These films were fabricated by layer-by-layer assembly with thicknesses at the sub-micrometer and micrometer scales. The OPL of the films can be calculated from the Fabry-Perot fringes on their reflection spectra. Based on the OPL of the films in dried and swollen conditions, we can also calculate the equilibrium swelling degree (SD_e_) of the films. The SD_e_ of the films can be tuned by a lot of external stimuli, including temperature, glucose, and fructose. The research shows that the film constantly shrinks as the temperature increases. However, when it is placed in a glucose or fructose solution, the films swell as the sugar concentration increases, and all the patterns of the films’ responses to multiple stimuli are linearly correlated, which is highly desirable for an ideal sensor because it leads to a simple calibration and constant sensitivity and precision over the entire linear range. More importantly, the speed of which the films respond to glucose or fructose is quite fast, with characteristic response times of 45 s and 7 s, respectively. These quick response films may have potential in real-time, continuous glucose monitoring.

A weakness of the present design is that the signal strength in response to glucose at a physiological pH is too low for subsequent practical application. We are now working to optimize the composition of the film to improve its responsiveness to glucose at a physiological pH.

## Figures and Tables

**Table 1:** Synthesis and characterization of P(DMAA-3-AAPBA) copolymers with different AAPBA content.

Entry No.	Feed Composition (mol%)		Copolymer Composition (mol%) ^a^		Mn ^b^	Mw ^b^
	AAPBA	DMAA	AAPBA	DMAA		
1	5%	95%	5%	95%	2.6 × 10^4^	3.2 × 10^4^
2	10%	90%	11%	89%	3.0 × 10^4^	4.5 × 10^4^
3	15%	85%	15%	85%	2.2 × 10^4^	4.2 × 10^4^

^a^: determined by ^1^H NMR. ^b^: determined by GPC.

## Data Availability

Not applicable.

## Supplementary Materials

The following supporting information can be downloaded at: https://www.mdpi.com/article/10.3390/polym15091998/s1, Figure S1: Experimental setup for film swelling study.

## References

[1] Tai Y., Chang W., Wan D., Chang Y., Ko F. (2021). A novel electronic assay based on a sol-gel transition reaction and a thin-film transistor of supramolecular hydrogels to detect alkaline phosphatase activity. Sens. Actuators B Chem..

[2] Kuroda N., Tounoue Y., Noguchi K., Shimasaki Y., Inokawa H., Takano M., Shinkai S., Tamaru S. (2020). Guest-responsive supramolecular hydrogels expressing selective sol-gel transition for sulfated glycosaminoglycans. Polym. J..

[3] Miyata T., Asami N., Uragami T. (2009). Structural design of stimuli-responsive bioconjugated hydrogels that respond to a target antigen. J. Polym. Sci. Part B Polym. Phys..

[4] Miyata T., Jige M., Nakaminami T., Uragami T. (2006). Tumor marker-responsive behavior of gels prepared by biomolecular imprinting. Proc. Natl. Acad. Sci. USA.

[5] Miyata T., Asami N., Uragami T. (1999). A reversibly antigen-responsive hydrogel. Nature.

[6] Hibbins A.R., Kumar P., Choonara Y.E., Kondiah P., Marimuthu T., Du T., Pillay V. (2017). Design of a Versatile pH-Responsive Hydrogel for Potential Oral Delivery of Gastric-Sensitive Bioactives. Polymers.

[7] Kim A., Mujumdar S.K., Siegel R.A. (2014). Swelling Properties of Hydrogels Containing Phenylboronic Acids. Chemosensors.

[8] Gawel K., Barriet D., Sletmoen M., Stokke B. (2010). Responsive hydrogels for label-free signal transduction within biosensors. Sensors.

[9] Li Z., Guan J. (2011). Thermosensitive hydrogels for drug delivery. Expert Opin. Drug Deliv..

[10] Stuart M.A., Huck W.T., Genzer J., Müller M., Ober C., Stamm M., Sukhorukov G., Szleifer I., Tsukruk V., Urban M. (2010). Emerging applications of stimuli-responsive polymer materials. Nat. Mater..

[11] Guan Y., Zhang Y. (2013). Boronic acid-containing hydrogels: Synthesis and their applications. Chem. Soc. Rev..

[12] Chen M., Zhang Y., Jia S., Zhou L., Guan Y., Zhang Y. (2015). Photonic Crystals with a Reversibly Inducible and Erasable Defect State Using External Stimuli. Angew. Chem. Int. Ed..

[13] Buenger D., Topuz F., Groll J. (2012). Hydrogels in sensing applications. Prog. Polym. Sci..

[14] Xiang Y., Xian S., Ollier R.C., Yu S., Su B., Pramudya I., Webber M. (2022). Diboronate crosslinking: Introducing glucose specificity in glucose-responsive dynamic-covalent networks. J. Control. Release.

[15] Bischofberger I., Calzolari D.C.E., De Los Rios P., Jelezarov I., Trappe V. (2015). Hydrophobic hydration of poly-N-isopropyl acrylamide: A matter of the mean energetic state of water. Sci. Rep..

[16] Halperin A., Kröger M., Winnik F.M. (2015). Poly(N-isopropylacrylamid)-Phasendiagramme: 50 Jahre Forschung. Angew. Chem..

[17] Zhang J., Huang S., Xue Y., Zhuo R. (2005). Poly(N-isopropylacrylamide) Nanoparticle-Incorporated PNIPAAm Hydrogels with Fast Shrinking Kinetics. Macromol. Rapid Commun..

[18] Zhang J., Zhao N., Zhang M., Li Y., Chu P., Guo X., Di Z., Wang X., Li H. (2016). Flexible and ion-conducting membrane electrolytes for solid-state lithium batteries. Nano Energy.

[19] Zhang Y., Guan Y., Zhou S. (2006). Synthesis and volume phase transitions of glucose-sensitive microgels. Biomacromolecules.

[20] Zhang X., Guan Y., Zhang Y. (2011). Ultrathin Hydrogel Films for Rapid Optical Biosensing. Biomacromolecules.

[21] Kuenstler A.S., Lahikainen M., Zhou H., Xu W., Priimagi A., Hayward R. (2020). Reconfiguring Gaussian Curvature of Hydrogel Sheets with Photoswitchable Host-Guest Interactions. ACS Macro Lett..

[22] Jia Y., Sun J., Yu D., Wang L., Campbell A., Fan H., Sun H. (2023). Light and hydrogen peroxide dual-responsive DNA interstrand crosslink precursors with potent cytotoxicity. Bioorganic Chem..

[23] Ding H., Zhang X.N., Zheng S.Y., Song Y., Wu Z., Zheng Q. (2017). Hydrogen bond reinforced poly(1-vinylimidazole-co-acrylic acid) hydrogels with high toughness, fast self-recovery, and dual pH-responsiveness. Polymer.

[24] Wang D., Hao J. (2013). Multiple-stimulus-responsive hydrogels of cationic surfactants and azoic salt mixtures. Colloid Polym. Sci..

[25] Robin S. (2018). Multistimuli-Responsive Foams Using an Anionic Surfactant. Langmuir.

[26] Ren J., Liu Y., Wang Z., Chen S., Ma Y., Wei H., Hua W., Lu S. (2022). An anti-swellable hydrogel strain sensor for underwater motion detection. Adv. Funct. Mater..

[27] Liu Y., Zhang Y., Guan Y. (2009). New polymerized crystalline colloidal array for glucose sensing. Chem. Commun..

[28] Matsumoto A., Kurata T., Shiino D., Kataoka K. (2004). Swelling and Shrinking Kinetics of Totally Synthetic, Glucose-Responsive Polymer Gel Bearing Phenylborate Derivative as a Glucose-Sensing Moiety. Macromolecules.

[29] Ge G., Mandal K., Haghniaz R., Li M., Xiao X., Carlson L., Jucaud V., Dokmeci M., Ho G., Khademhosseini A. (2023). Deep Eutectic Solvents-Based Ionogels with Ultrafast Gelation and High Adhesion in Harsh Environments. Adv. Funct. Mater..

[30] Ben-Moshe M., Alexeev V.L., Asher S.A. (2006). Fast responsive crystalline colloidal array photonic crystal glucose sensors. Anal. Chem..

[31] Wojtecki R.J., Meador M.A., Rowan S.J. (2011). Using the dynamic bond to access macroscopically responsive structurally dynamic polymers. Nat. Mater..

[32] Xiao X., Zheng W., Zhao Y., Li C. (2023). Visible light responsive spiropyran derivatives based on dynamic coordination bonds. Chin. Chem. Lett..

[33] Suzuki N., Takahashi A., Ohishi T., Goseki R., Otsuka H. (2018). Enhancement of the stimuli-responsiveness and photo-stability of dynamic diselenide bonds and diselenide-containing polymers by neighboring aromatic groups. Polymer.

[34] Hong S.H., Kim S., Park J.P., Shin M., Kim K., Ryu J., Lee H. (2018). Dynamic Bonds between Boronic Acid and Alginate: Hydrogels with Stretchable, Self-Healing, Stimuli-Responsive, Remoldable, and Adhesive Properties. Biomacromolecules.

[35] Fan X., Zhang L., Dong F., Liu H., Xu X. (2023). Room-temperature self-healing polyurethane–cellulose nanocrystal composites with strong strength and toughness based on dynamic bonds. Carbohydr. Polym..

[36] Zeng W., Jin Y., Li Y., Zhou R., Shi L., Bai L., Shang X., Li J. (2023). Ambient temperature self-healing waterborne polyurethane based on dynamic ditelluride bonds with recyclable and antibacterial functions. Prog. Org. Coat..

[37] Zhou Y., Zeng G., Zhang F., Li K., Li X., Luo J., Li J., Li J. (2023). Design of tough, strong and recyclable plant protein-based adhesive via dynamic covalent crosslinking chemistry. Chem. Eng. J..

[38] Cao J., Zhao X., Ye L. (2022). Super-strong and anti-tearing poly(vinyl alcohol)/graphene oxide nano-composite hydrogels fabricated by formation of multiple crosslinking bonding network structure. J. Ind. Eng. Chem..

[39] Wang X., Cao L., Xu C., Fan B., Lin Z., Li W., Zhang P. (2022). Novel dual dynamic boronate ester bond regulated bio-based polymer with rapid self-healing and multiple recyclability. Ind. Crops Prod..

[40] Shiomori K., Ivanov A.E., Galaev I.Y., Kawano Y., Mattiasson B. (2004). Thermoresponsive Properties of Sugar Sensitive Copolymer ofN-Isopropylacrylamide and 3-(Acrylamido)phenylboronic Acid. Macromol. Chem. Phys..

[41] Li Q., Guan Y., Zhang Y. (2018). Thin hydrogel films based on lectin-saccharide biospecific interaction for label-free optical glucose sensing. Sens. Actuators B Chem..

[42] Zhao Y., Gu J., Jia S., Guan Y., Zhang Y. (2016). Zero-order release of polyphenolic drugs from dynamic, hydrogen-bonded LBL films. Soft Matter.

[43] Zhao Y., Yuan Q., Li C., Guan Y., Zhang Y. (2015). Dynamic Layer-by-Layer Films: A Platform for Zero-Order Release. Biomacromolecules.

[44] Zhou L., Chen M., Guan Y., Zhang Y. (2014). Multiple responsive hydrogel films based on dynamic Schiff base linkages. Polym. Chem..

[45] Ding Z., Guan Y., Zhang Y., Zhu X. (2009). Layer-by-layer multilayer films linked with reversible boronate ester bonds with glucose-sensitivity under physiological conditions. Soft Matter.

[46] Omer R.A., Hughes A., Hama J.R., Wang W., Tai H. (2015). Hydrogels from dextran and soybean oil by UV photo-polymerization. J. Appl. Polym. Sci..

[47] Guan Y., Yang S., Zhang Y., Xu J., Han C., Kotov N. (2006). Fabry-Perot Fringes and Their Application To Study the Film Growth, Chain Rearrangement, and Erosion of Hydrogen-Bonded PVPON/PAA Films. J. Phys. Chem. B.

[48] Buwalda S.J., Boere K.W., Dijkstra P.J., Feijen J., Vermonden T., Hennink W. (2014). Hydrogels in a historical perspective: From simple networks to smart materials. J. Control. Release.

[49] Matsumoto A., Yoshida R., Kataoka K. (2004). Glucose-responsive polymer gel bearing phenylborate derivative as a glucose-sensing moiety operating at the physiological pH. Biomacromolecules.

[50] Nishiyabu R., Kubo Y., James T.D., Fossey J.S. (2011). Boronic acid building blocks: Tools for sensing and separation. Chem. Commun..

[51] Alexeev V.L., Sharma A.C., Goponenko A.V., Das S., Lednev I.K., Wilcox C.S., Finegold D.N., Asher S.A. (2003). High ionic strength glucose-sensing photonic crystal. Anal. Chem..

