# Supplementary figures and images for: Multiple Responsive Hydrogel Films Based on Dynamic Phenylboronate Bond Linkages with Simple but Practical Linear Response Mode and Excellent Glucose/Fructose Response Speed
Source: Polymers (Basel). 2023 Apr 23;15(9):1998. doi: 10.3390/polym15091998 (PMC10181213; doi:10.3390/polym15091998)

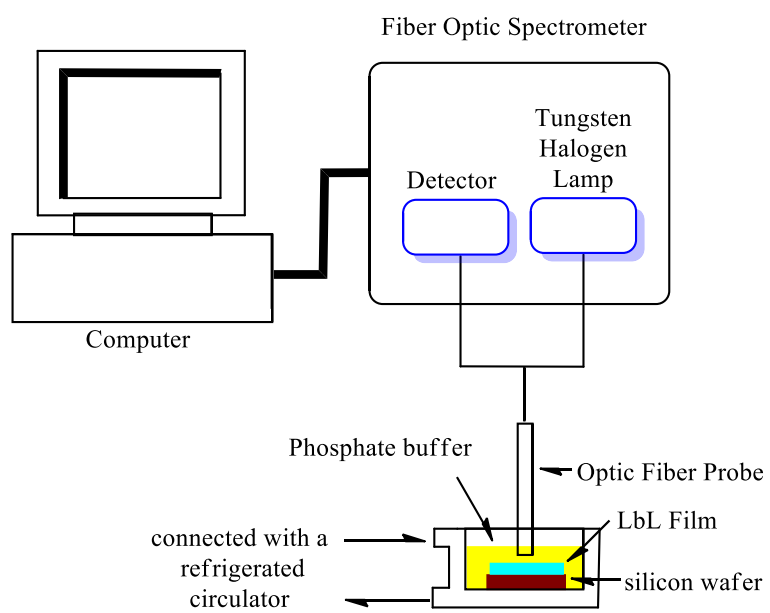

Figure S1. Experimental setup for film swelling study.

Supplement: Supplementary file 1 [file polymers-15-01998-s001.zip › polymers-2352997-supplementary.pdf]
